# Virtual Screening Based on Machine Learning Explores Mangrove Natural Products as KRAS^G12C^ Inhibitors

**DOI:** 10.3390/ph15050584

**Published:** 2022-05-08

**Authors:** Lianxiang Luo, Tongyu Zheng, Qu Wang, Yingling Liao, Xiaoqi Zheng, Ai Zhong, Zunnan Huang, Hui Luo

**Affiliations:** 1 The Marine Biomedical Research Institute, Guangdong Medical University, Zhanjiang 524023, China; 2 The Marine Biomedical Research Institute of Guangdong Zhanjiang, Zhanjiang 524023, China; 3 The First Clinical College, Guangdong Medical University, Zhanjiang 524023, China; dzheng@gdmu.edu.com (T.Z.); wang15728276376@hotmail.com (Q.W.); lyl212608@gdmu.edu.cn (Y.L.); zxqzxq@gdmu.edu.cn (X.Z.); zhongai@gdmu.edu.cn (A.Z.); 4 School of Pharmacy, Guangdong Medical University, Dongguan 523808, China; 5 Key Laboratory of Big Data Mining and Precision Drug Design of Guangdong Medical University, Dongguan 523808, China

**Keywords:** mangrove natural products, KRAS^G12C^, machine learning, molecular docking, drug discovery, virtual screening, molecular dynamics

## Abstract

Mangrove secondary metabolites have many unique biological activities. We identified lead compounds among them that might target KRAS^G12C^**.** KRAS is considered to be closely related to various cancers. A variety of novel small molecules that directly target KRAS are being developed, including covalent allosteric inhibitors for KRAS^G12C^ mutant, protein–protein interaction inhibitors that bind in the switch I/II pocket or the A59 site, and GTP-competitive inhibitors targeting the nucleotide-binding site. To identify a candidate pool of mangrove secondary metabolic natural products, we tested various machine learning algorithms and selected random forest as a model for predicting the targeting activity of compounds. Lead compounds were then subjected to virtual screening and covalent docking, integrated absorption, distribution, metabolism and excretion (ADME) testing, and structure-based pharmacophore model validation to select the most suitable compounds. Finally, we performed molecular dynamics simulations to verify the binding mode of the lead compound to KRAS^G12C^. The lazypredict function package was initially used, and the Accuracy score and F1 score of the random forest algorithm exceeded 60%, which can be considered to carry a strong ability to distinguish the data. Four marine natural products were obtained through machine learning identification and covalent docking screening. Compound **44** and compound **14** were selected for further validation after ADME and toxicity studies, and pharmacophore analysis indicated that they had a favorable pharmacodynamic profile. Comparison with the positive control showed that they stabilized switch I and switch II, and like MRTX849, retained a novel binding mechanism at the molecular level. Molecular dynamics analysis showed that they maintained a stable conformation with the target protein, so compound **44** and compound **14** may be effective inhibitors of the G12C mutant. These findings reveal that the mangrove-derived secondary metabolite compound **44** and compound **14** might be potential therapeutic agents for KRAS^G12C^.

## 1. Introduction

Plants are an important source of drugs and many compounds extracted from plants have been shown to have excellent medicinal properties [1]. In China and India, drugs extracted from plants have been widely put into use. For example, ethyl acetate compounds obtained from the flowers of Cassia fistula [2]. Mangroves are widely distributed in tropical and subtropical beach areas and grow a variety of plants with rich medicinal value, such as Scyphiphora and Clerodendruminerme [3]. Additionally, mangroves have unique biochemical properties that produce a large number of novel natural products and complex skeletons, and various extracts of mangrove plants have been used to treat tumors and bacterial infections [4]. Mangrove secondary metabolite extracts contain a large number of medicinal compounds similar to tannins, steroids, triterpenes, saponins, etc. [5]. These medicinal compounds play a unique role in the fight against human and animal pathogens.

KRAS targets have long been considered typically non-targetable targets in drug discovery, circulating in active and inactive GTPase [6,7,8]. RAS is a GTPase that cycles between GTP-bound (active) state and GDP-bound (inactive) forms through the actions of guanine nucleotide exchange factors (GEFs) and GTPase-activating proteins (GAPs). In the active state, KRAS can maintain affinity with many proteins, among which RAF and PI3K pathways may be the most typical [9,10,11]. Although the KRAS protein is closely associated with cancer, recent studies have shown that the KRAS protein can cause oncogenic mutations around activation sites (e.g., G12C, G12D, etc.), leading to downstream RAFs and overexpression of PI3K proteins. This leads to a range of cancerous lesions and over-proliferation of cancer cells [12]. In KRAS mutations, mutations in Cys12 cause the KRAS^G12C^ protein to lose its inherent catalytic activity and at the same time to lose the GTPase-activated protein (GAP). The enhanced catalytic effect leads to the activation of its structure and disrupts the inactive state of KRAS, causing cancer cells to promote proliferation. Lung cancer is the leading cause of cancer deaths in Western countries. In the course of the current study, although we have made substantial progress in treating genetic subtypes (e.g., patients with EGFR mutations or ALK-translocated lung cancer), the most common (30% genetically defined subtypes) and effective treatment strategies are still lacking. Cys12 mutations caused by codon 12 mutations account for nearly 50% of patients with KRAS mutations [13,14,15]. Therefore, drugs that target KRAS^G12C^ may have important therapeutic effects. Although KRAS is one of the first oncogenes to be discovered, it has two features that make it nearly impossible to be suppressed. 1. KRAS binds to GTP and GDP with a dermo maline affinity, which makes it difficult to develop nucleotide-based inhibitors [16]. 2. The hydrophobic pocket of KRAS is shallow, resulting in the insufficient affinity of the compound with the KRAS protein, resulting in off-target effects and increasing the difficulty of finding high-affinity allosteric inhibitors [17].

In 2013, Shokat and his colleagues used a new strategy for Cys12 for KRAS^G12C^ [18]. They suggested that covalent bonds formed with Cys12 residue could interfere with the activity of KRAS^G12C^, thereby locking it into an inactive state bound to GTP, thereby downregulating the downstream signaling pathway. After that, the researchers developed a series of covalent inhibitors (ARS1620, AMG510, MRTX849) [19,20,21,22], that passed with Cys12 and switch II (amino acids 60–68) binds and occupies an allogeneic pocket on KRAS, and in these findings, the hydrophobic fraction of the new inhibitor penetrates switch II. In the allogeneic pockets below the ring area (residue His95, Tyr96, Gln99), these inhibitors are highly selective for the state in which KRAS^G12C^ binds to GTP and can maintain the inactivity of the KRAS^G12C^ protein in the formation of its covalent complex. 

**Figure 1:**
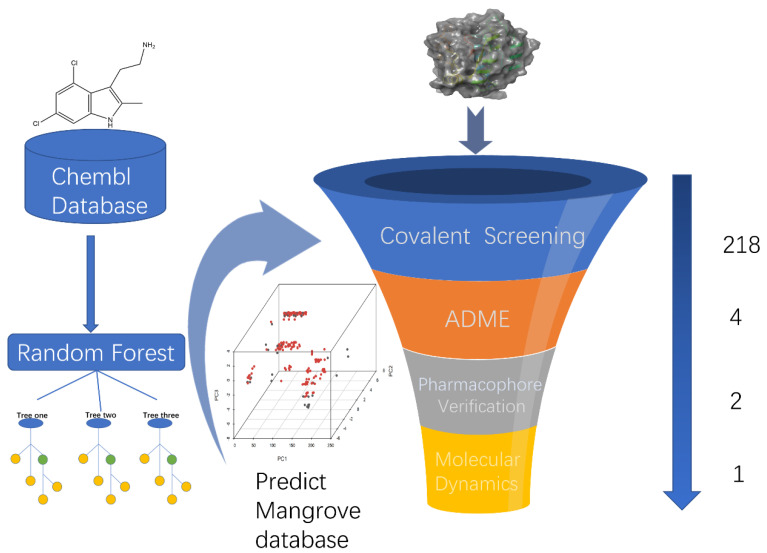
A virtual screening workflow (VSW) was used to identify molecules that hit KRAS^G12C^. A workflow overview of machine learning, covalent screening, elimination, and toxicity (ADMET) approaches for pharmacophore validation and MD simulations.

To begin with, we performed preliminary virtual filtering based on ligand structures. Then, we constructed training and test sets based on a selection of KRAS^G12C^ inhibitors from the ChEMBL library and trained a random forest classifier to prospectively predict a library of candidate compounds that were then predicted to be active by covalent screening. More effective chemical structures than positive compounds were screened by comprehensive evaluation of docking score and MM-GBSA score, and lead compounds were selected by ADME toxicity analysis. A compound adapted to KRAS^G12C^ was selected and it can be considered that it may be an inhibitor of KRAS^G12C^. Under this process, we can predict the lead components that may target KRAS^G12C^ in the mangrove natural product library under the algorithm that is most suitable for the training set and then select the compounds with better docking effects than the positive control for ADME property analysis to reduce false positives in ADME screening as much as possible. Pharmacophore validation demonstrated that the lead compound and the positive control had more common features, and kinetics further validated the binding activity of the selected compounds (Figure 1).

## 2. Result

### 2.1. Candidate Compound Library Data

**Figure 2:**
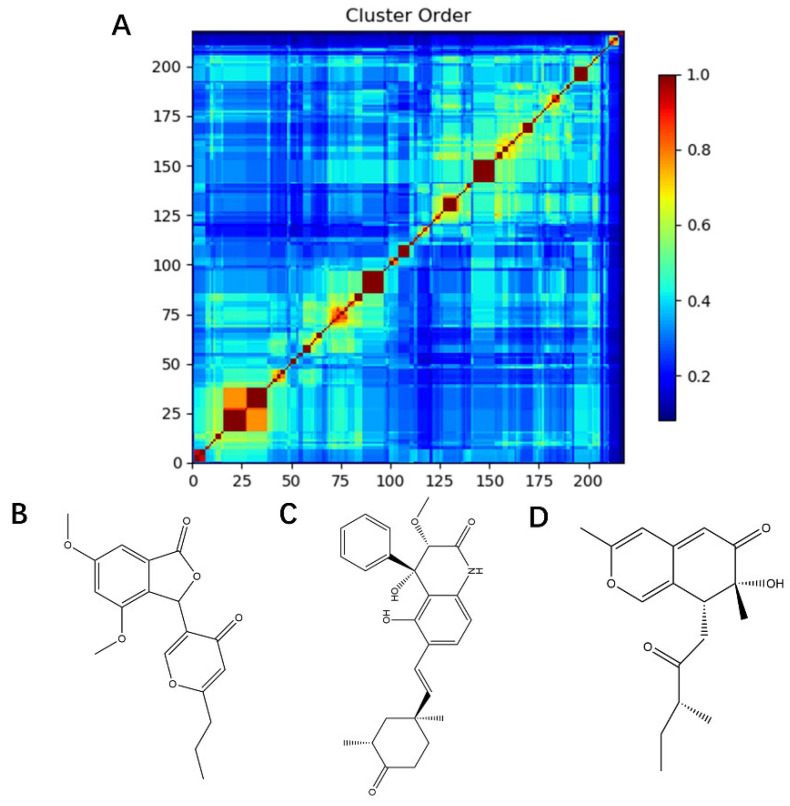
Clustering and typical frameworks of clusters in mangrove secondary metabolite library. (**A**) Clustering of clusters in the mangrove secondary metabolite library; (**B**–**D**) typical frameworks in the candidate compound library.

The dataset for external use was from the ChEMBL database, and all data excluded compounds that did not have semi-concentration inhibition activity. To obtain the distribution of compound species in the mangrove secondary metabolite library, we calculated the structural similarity score (volume score) between each compound in the dataset. The volume score is between 0 and 1, and a higher value indicates higher structural similarity. The following figure reports the volume score between the two compounds. The two-volume score was obtained by fractional normalization based on the backbone of the first or second compound. Additionally, the result demonstrates the cluster analysis of the corresponding volume score sizes for different compounds in the mangrove natural product library, where most compounds have a similarity score of less than 0.4. Figure 2A reveals three sets, compounds **0**–**50**, compounds **50**–**100**, the high similarity of compounds **125**–**200** may be due to the presence of co-backbone structures in the mangrove secondary metabolite pool (Figure 2B–D). 

### 2.2. Machine Learning Models

**Figure 3:**
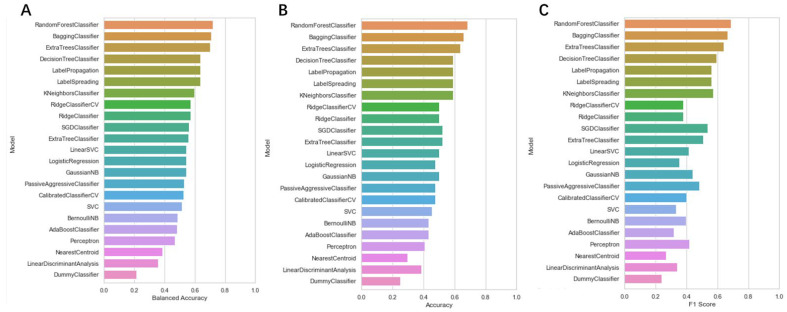
The accuracy of each machine learning algorithm trained using the lazypredict package. (**A**) Balanced Accuracy value of each machine learning classifier; (**B**) Accuracy value of each machine learning classifier; (**C**) F1 score value of each machine learning classifier.

To screen the mangroves in the laboratory to find inhibitors that can better target KRAS^G12C^, we used machine learning technology to better predict the activity of these compounds on the target protein. Machine learning models were developed using the random forest model and the various algorithms included in the lazypredict package. All data were cross-validated by 10x, and from our result, it is clear that different predictive statistics for all machine learning algorithms were implemented using training data alone. At a threshold of 6.5um, the random forest algorithm performed best among all candidate classification algorithms, with the highest Accuracy and F1 score, indicating that the random forest algorithm had the best-fit value. (Figure 3) Therefore, we chose the random forest classification algorithm in Weka (version 3.8) to train the pubchem molecular fingerprint and analyze its 882 features. However, in real-world analysis, many features are complex and have noise implications for the analysis. Therefore, with the Rank and CfsSubsetEval modules, we removed features that were not related to structure–activity effects and finally analyzed the remaining 404 features, improving the performance of the random forest algorithm. Consequently, from a variety of classifiers, we screened and applied the random forest classifier that best fit the dataset, showing its good discriminative power.

### 2.3. Random Forest Classification Model

**Figure 4:**
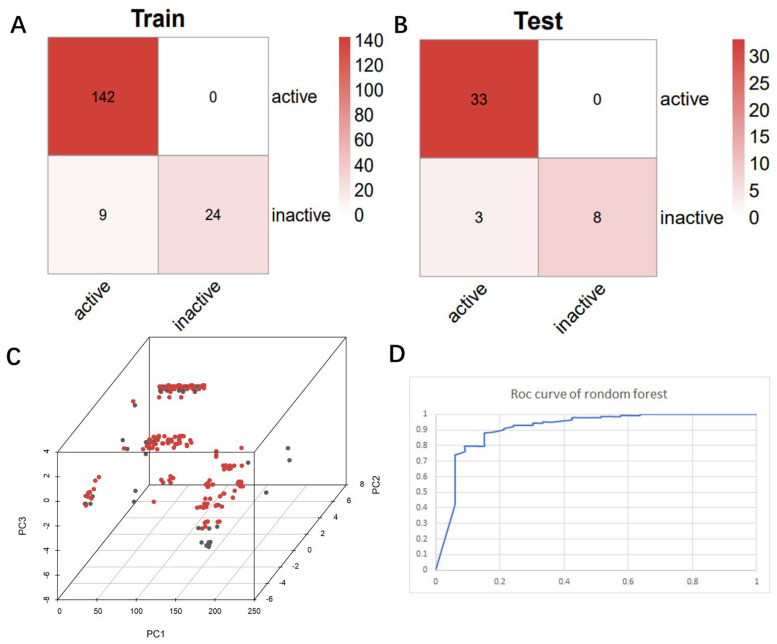
Chaos matrix and roc curve of the constructed random forest classifier and the chemical space of the candidate mangrove compound library. (**A**) Confusion matrix of random forest classifier to distinguish the training set; (**B**) confusion matrix of random forest classifier to distinguish the test set; (**C**) chemical space of the candidate compound library; (**D**) ROC curve of the random forest classifier.

After identifying the machine learning classifier likely to best fit the ChEMBL dataset, we performed a new round of parameter tuning for the random forest algorithm, which helped to better identify new molecules with similar properties that bind to the target protein. The descriptor set obtained by the feature selection method was used to establish a classification model, and the machine learning algorithm of random forest classification was used to evaluate the molecular descriptor set to be selected in detail. Confusion matrices are visualization tools used in machine learning to show accuracy assessments in supervised learning. The records in the dataset were summarized in matrix form based on the two criteria for the actual category and the classification judgments made by the classification model. The random forest model was suitable for both true positives and true negatives (Figure 4A,B), with a false positive number of 9 and a false negative number of 24 in the training set. The number of false positives in the test set is 3 and the number of false negatives is 8. Compared with true positives and true negatives in the matrix, good classification effects can be shown. A good binary classification model usually has good sensitivity and resolution, accuracy, and a larger area under ROC. If both sensitivity and resolution are high, the accuracy will be biased towards the highest value. Figure 4D shows the area under the ROC curve of the machine learning algorithm. It can be seen that it has a high ROC value (ROC = 0.965), which partly indicates that it has a strong ability to distinguish between molecular descriptors. Additionally, it is sufficient for the activity differentiation of mangrove natural product libraries. In Table 1 and Table 2, the predicted values, recall values, F-scores, and MCC scores of the active and inactive compound classifiers are given, and the values of the average model after the mixture of the two are given. The predicted, recall values, and F-scores of the active and inactive compounds in the training set were all close to 1, showing excellent sensitivity, with an average model MCC value of 0.825. While the average model in the test set has better overall statistics, a better balance is achieved between recall and specificity.

### 2.4. Chemical Space

The chemical space of the mangrove secondary metabolite library is highly diverse, consisting of aldehydes, alcohols, and esters. The data set for this study was established using compounds extracted from secondary metabolites of mangroves collected in published papers. The data set was constructed with Schrodinger software, and a total of 281 molecules from different bacterial groups were collected. Principal component analysis was performed on secondary metabolic natural products in mangrove forests (Figure 4C). When analyzed using molecular fingerprint descriptors, it can be seen that KRAS^G12C^ is spatially well-arranged, active and inactive molecules are well-arranged and are located in large clusters with wider distributions. Splitting the test data in the compound library into the 80% training dataset and 20% test dataset shows the considerable overlap between the two sets (Figure 4C), which shows that the classifier is validated based on COM with similar intervals.

### 2.5. Prediction of Prospects

Candidate compounds were scored by a random forest model and compounds labeled as active were selected for subsequent docking experiments. From this point of view, compounds are docked based on reliability, which may provide a degree of confidence for these predictions. 

### 2.6. Docking

**Figure 5:**
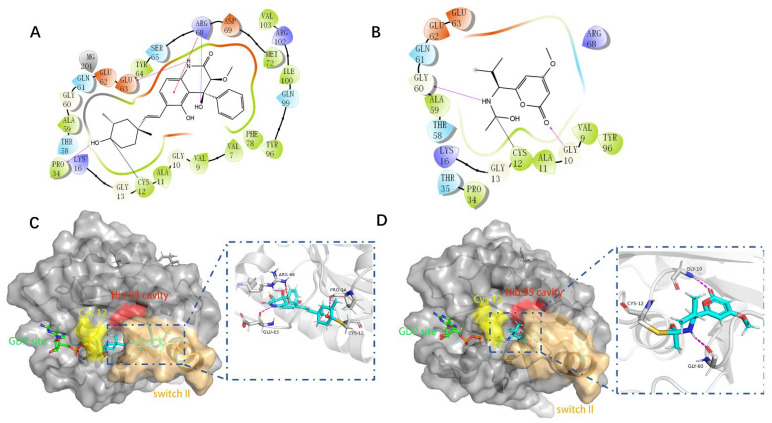
The most pharmaceutically available compound **44** with compound **14** and the KRAS^G12C^ docking structure diagram. (**A**) Two-dimensional binding mode of compound **44** and protein complex; (**B**) two-dimensional binding mode of compound **14** and protein complex; (**C**) three-dimensional binding mode of compound **44** and protein complex; (**D**) three-dimensional binding modes of compound and protein complexes. The purple sticks are hydrogen bonds and the red sticks are ΠΠ cation interactions.

Molecular docking can better reveal how compounds bind to targets. The selected lead compounds were covalently docked in the Schrödinger Suite 18.4. After the ligand minimization step, the interaction energy of each compound at each docking position was calculated. The compounds with better performance than the positive control MRTX849 score were selected to show the most favorable conformation. It can be seen that the final selected compound is better combined in the preset binding pocket and wrapped tightly, so it is judged that its off-target possibility is not high. Figure 5 shows 2D and 3D interaction patterns of compounds **44** and **14** docking. Among them, the 2D interaction diagram of compound **44** can be seen in Figure 5A and the 3D docking mode diagram in Figure 5C. For compound **14**, its two-dimensional interaction diagram and three-dimensional docking model diagram are shown in Figure 5B,D. It can be seen that both compounds are linked to the H95 cryptocodon of KRAS^G12C^ and form an irreversible covalent bond with Cys12. Both compounds are connected to the switch II region, where the KRAS^G12C^ protein can be inactivated by regulation. Compound **14** had a solid docking conformation by interacting with Gly60 to form a hydrogen bond, forming a hydrogen bond with Gly10. Compound **44** is firmly present by forming the ΠΠ interaction with Arg68 and a hydrogen bond interaction and forming hydrogen bonds with Peo34, surrounded by hydrophobic bonds in the pocket. The results of validation and docking are consistent, which is qualitatively expected for an inhibitor of KRAS^G12C^. The RMSD values of compound **44** and compound **14** with the best docking effect are 7.9196 and 9.1083, respectively, which are in line with the docking agreement among all the compounds with better docking effects, showing that the overlap with MRTX849 fluctuates less (Table 3).

### 2.7. MM-GBSA

**Figure 6:**
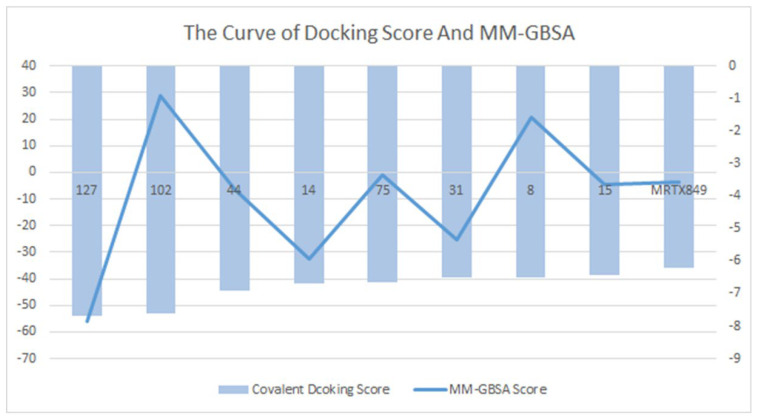
Compounds that were identified as active by random forest classifiers and were superior to positive controls in covalent screening with KRAS^G12C^ protein docking results and MM-GBSA results.

Binding free energies calculated by molecular mechanics generalized born surface area (MM-GBSA) indicate that the compensation between binding enthalpy and entropy plays a crucial role in drug–protein binding. For the alternative lead compound, calculations were also performed in the MM-GBSA module in the Schrödinger Suite 18.4. The values of MM-GBSA for each compound were obtained, and the compound superior to the positive control MRTX849 was selected (Figure 6). We describe the surrounding kinetic environment by analyzing the conformation of protein ligands, but the calculations are too complex to be easily controlled. We selected conformation by analyzing intermolecular MM-GBSA scores and binding fractions, which not only focus on binding but also visualize it by a fraction. The MRTX849 compound has an MM-GBSA score of −3.58, and compound **127** (score = −55.97), compound **44** (score = −6.35), compound **14** (score = −32.68), compound **31** (score = −25.13), and compound **15** (score = −4.43) are higher than the MRTX849, indicating that these compounds may have a better conformation than the positive control.

### 2.8. ADME

**Figure 7:**
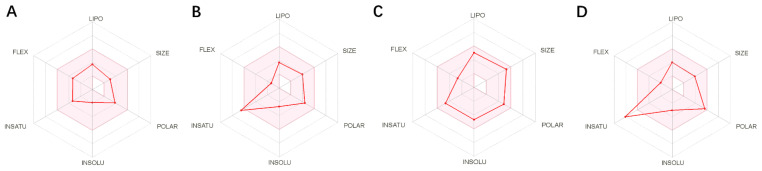
ADME properties of compounds obtained after the covalent screening. (**A**) ADME properties of compound **14**; (**B**) ADME properties of compound **31**; (**C**) ADME properties of compound **44**; (**D**) ADME properties of compound **127**.

**Figure 8:**
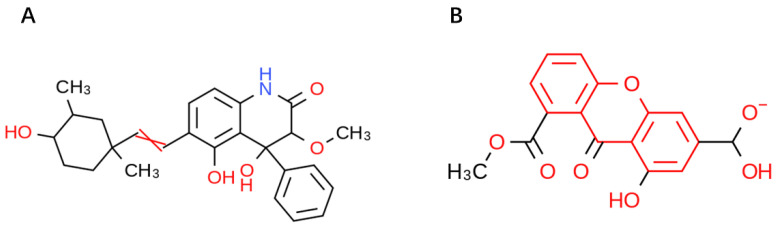
Toxicity alerts for compounds **44** and **31** are displayed in red font. Toxicity alerts for compounds **44** and **31** are displayed in red font. (**A**) Toxicity alert for compound **44**; (**B**) toxicity alert for compound **31**.

ADME (Absorption, Distribution, Metabolism, and Excretion) is a key aspect for predicting the pharmacodynamics of the molecule under study which could be used as a future lead molecule for drug development. Swiss-ADME is a website (https://www.swissadme.ch, accessed on 17 December 2021) that allows users to draw individual ligands or drug molecules or contains molecules from pubchem smiles data and provides information such as fat solubility (iLOGP, XLOGP3, WLOGP, MLOGP, SILICOS-IT, Log Po/w), Water Soluble Log S (ESOL, ALI, SILICOS-IT), drug-like rules (Lipinski, Ghose, Veber), and other parameters. ADME prediction studies for design compounds are shown in Table 4. Swiss-ADME is based in part on Lipinski, Ghose, Veber, Egan, and the five different rules identified by Muegge give the physicochemical properties of a possible oral drug candidate [23,24,25,26]. The logarithmic S reference values for medium soluble and highly soluble molecules are −4 to −6 and −2~−4, respectively. Based on the results, all molecules are classified as medium soluble and highly soluble. ADME drug capability assessment was performed on four selected compounds, where compounds **127** and **31** violated Lipinsky’s rule of five, but compound **44** and compound **14** exhibited good ADME properties (Figure 7) and were reserved for the next evaluation and submitted for bone toxicity analysis, where the benzene backbone of compound **31** showed stronger toxicity (Figure 8) (https://mcule.com/apps/toxicity-checker/, accessed on 19 January 2022). The next evaluation was skipped. All of these parameters infer that compounds **44** and **14** are close to a drug-like molecule. 

### 2.9. Pharmacophore Analysis

**Figure 9:**
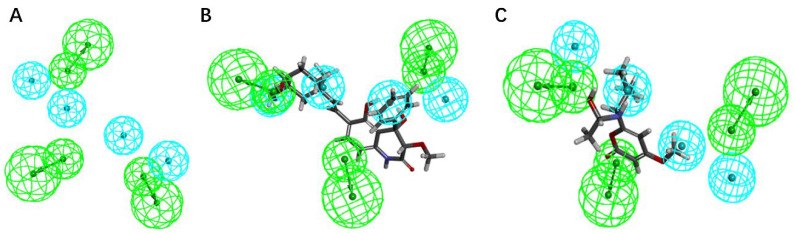
The pharmacophore model was generated based on the common features of the positive compounds and the matching status of the two candidate molecules with the model. (**A**) The common feature pharmacophore model; (**B**) the matching status of compound **44** and the model; (**C**) the matching status of compound **14** with the model.

During the process of virtual screening, the pharmacophore model can be used to characterize the active conformation of the ligand molecule by conformational search and molecular superposition, and the possible mode of action between the receptor and the ligand molecule can be deduced and explained accordingly. Based on the ranking and scoring results of the pharmacophores given by the platform (Table 5), the best pharmacophores we selected (rank score = 52.937) have four hydrophobic characteristics and three hydrogen bond receptors. The results confirm that both drug candidates match the selected model, with compound **44** being the best match to the pharmacophore, matching the three hydrophobic interaction features and one hydrogen bond acceptor feature (green) of the model (Figure 9). It can be assumed that both compounds are more similar to the known inhibitors in terms of distribution of spatial pharmacodynamic curves.

### 2.10. Root Mean Square Deviation (RMSD) Analysis

To obtain the equilibration time for each simulated protein–ligand complex during the MD simulation, the RMSD of the skeleton was calculated. RMSD plots are typically used to evaluate the time it takes for a system to reach structural balance and to estimate the duration of running a simulation. RMSD is an important parameter for estimating changes or changes in molecular conformation. Due to sudden changes in structural conditions, the RMSD value of analog complexes, including references, increases suddenly, which is related to protein crystallization. The latter effect is to be expected since, in the crystal structure, the protein is rigid, and when it dissolves in the tank it resumes its dynamic movement.

A complex system with a time frame x should have an *RMSD* that can be calculated from the following equation [27,28].

(1) $$RMSD_{x}=\sqrt{\frac{1}{N}\sum_{i=1}^{N}\left({r^{\prime }}_{i}\left(t_{x}\right)\right)-r_{i}\left(t_{ref}\right){)}^{2}}$$

Here, the $RMSD_{x}$ is the calculation of *RMSD* for the specific number of frames, N is the number of selected atoms; $t_{ref}$ is the reference or mentioned time, $r^{\prime }$ is the selected atom in the frame x after super imposing on the reference frame, and $t_{x}$ is the recording intervals.

### 2.11. Root Mean Square Volatility (RMSF) Analysis

**Figure 10:**
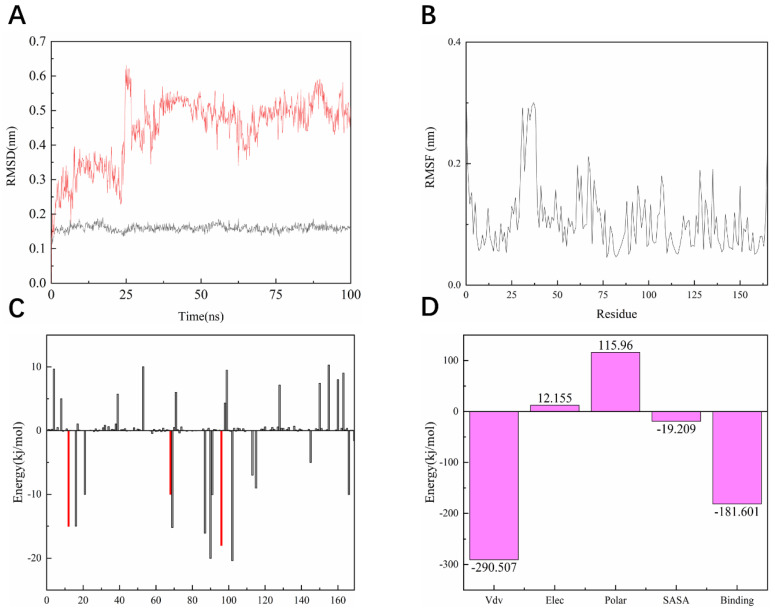
Dynamic simulation results. (**A**) RMSD values extracted from protein fit ligand of the protein-ligand docked complexes and ligand. RMSD plot of KRAS^G12C^ complex (red) and ligand 44 (black). (**B**) The RMSF graph of all complexes along with the protein during 100 ns MD simulation. RMSF plot of the KRAS^G12C^ complex (black). (**C**) Residue-wise decomposition of binding free energies obtained from the MM-PBSA analyses. Red bars indicate CYS-12, ARG-68, and TYR-96. (**D**) Binding energy of binding for the protein complexed with ligand 44.

**Figure 11:**
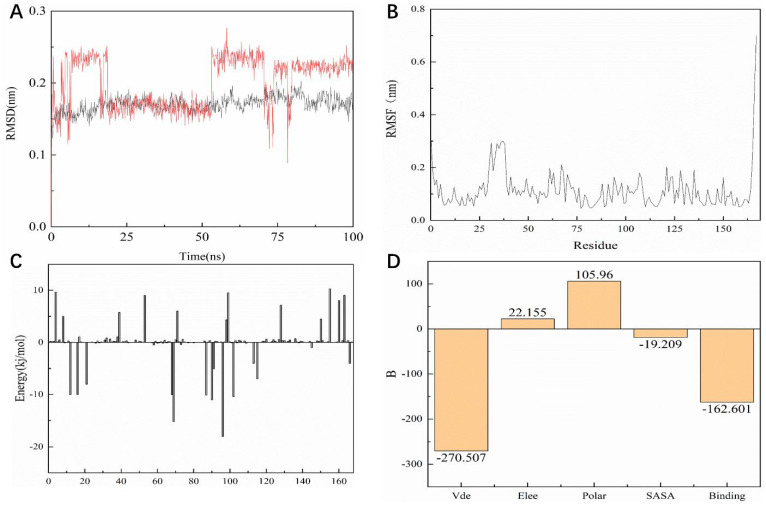
Dynamic simulation results. (**A**) RMSD values extracted from protein fit ligand of the protein-ligand docked complexes and ligand. RMSD plot of the KRAS^G12C^ complex (red) and ligand 14 (black). (**B**) The RMSF graph of all complexes along with protein during 100 ns MD simulation. RMSF plot of the KRAS^G12C^ complex (black). (**C**) Residue-wise decomposition of binding free energies obtained from the MM-PBSA analyses. (**D**) Binding energy of binding for the protein complexed with ligand 14.

As shown in Figure 10A, the entire KRAS^G12C^ system is in equilibrium in the first 62 ns of the simulated 100 ns (RMSD value is 0.52 nm), and then fluctuates to the RMSD of 0.50 nm after 62 ns and in equilibrium in the remaining 38 ns; the entire system can eventually be in equilibrium in this 100 ns without much fluctuation in the process. For the system of compound **14**, the RMSD of the system is finally stable at 0.23 nm and the RMSD of its ligand is stable at around 0.16 nm. However, in the 100 ns simulation process, it can be seen that there are four relatively long-term fluctuations. Interestingly, the system finally stabilizes and is 0.27 nm lower than the RMSD value of compound **44** (Figure 11A). To determine the deviation of the ligand from the initial posture and the degree of movement of the protein residues, the RMSF values of all sampled conformations during the 30 ns simulation were also calculated. RMSF fluctuates greatly, indicating that the residue is unstable; otherwise, the residue is stable. The RMSF of the residue is i ccalculated from the following equation [29]. 

As shown in Figure 10B, the RMSF range of the entire system is between 0.05 and 0.3 nm. From this numerical range, the flexibility of the entire complex system is relatively low, and each residue does not fluctuate too much. The RMSF value of the residue ranged from 0.05 to 0.15 nm, indicating that the binding site of compound **44** with the target fluctuated significantly and the binding was stable. The RMSF of another system is basically consistent with the overall surface line of RMSF of compound **44**. After visualization, the RMSF of compound **14** is significantly larger on the key residues Cys12, Arg68, and Tyr96. The flexibility of these residues shows that compound **14** is not as effective as compound **44** (Figure 11B).

### 2.12. MM/PBSA Analysis

For molecular mechanics, Poisson–Boltzmann surface area (MM/PBSA) is an efficient and reliable method for calculating the free energy of small inhibitors bound to their protein targets. In general, low binding energy values indicate that the binding between the ligand and the target is good, and the results of the g_mmpbsa are shown in Figure 10C,D. In Figure 10C we can see that for the key residues Cys12, Arg68, and Tyr96, the energy contribution of Cys12, Arg68, and Tyr96 is −15.05, −15.21, and −18.02 kj/mol, which also corresponds to the interaction forces shown in the molecular docking section results. It is shown that the interaction force acts in this system. In terms of the specific energy contribution of key residues Cys12, Arg68, and Tyr96, the energy value of compound 14 is not very good. These values are −10.03, −11.21, and −12.02 kj/mol, respectively (Figure 11C). In addition, in the energy decomposition of compound 44 and the target (Figure 10B), the total binding energy is −181.601 kj/mol, the energy of van der Waals is −290.5 kj/mol, the energy of electrostatic energy is 12.155 kj/mol, the energy of polarization is 115.96 kj/mol, and the final energy of SASA is −19.209 kj/mol. Overall, compound **44** binds very well to KRAS^G12C^. The total combined energy of compound **14** is −162.601 kj/mol, van der Waals energy is −290.5 kj/mol, electrostatic energy is 22.155 kj/mol, polarization energy is 105.96kj/mol, and the final energy of Sasa is −19.209 kj/mol (Figure 11D). 

## 3. Discussion

KRAS is the cancer gene with the most mutations in a single place and is the first to be identified to have a causal relationship with human cancer [30]. Mutations in KRAS are common among the three deadliest cancers: pancreatic, colorectal, and lung cancers [31]. Frequent mutations in the KRAS gene lead to increased demand for drug development, but due to its strong affinity with GTP and lack of deep hydrophobic pockets, the hydrogen bond formed by molecular docking has difficultly accurately anchoring its active pocket, which makes the development of corresponding small molecule inhibitors very difficult. However, recently, after the single mutation KRAS^G12C^ was identified as an inhibitor that could be used in clinical trials. There have been an increasing number of studies on KRAS^G12C^, the most common KRAS mutation in lung cancer individuals. 

In recent years, due to the low affinity of the binding form formed by the hydrogen bond and the limited binding efficiency of the active pocket, people have gradually begun to focus on covalent docking. Covalent bonds are directly connected to the target residues; thus, providing a more stable and higher affinity than hydrogen bonds. Therefore, in this paper, due to the special form of KRAS^G12C^ protein, we chose covalent screening as a way to find lead compounds, hoping to find new inhibitors from the mangrove natural product library through machine learning high-throughput screening. 

In this study, 281 published small molecules targeting KRAS^G12C^ were selected from the ChEMBL database and all of them were converted into pubchem molecular fingerprints. Molecular descriptors were evaluated with the lazypredict package in Python. The random forest classifier had higher AUC values among all the classifiers used. This means that compared to other machine learning methods, random forest outperforms all methods on the KRAS^G12C^ dataset, so the random forest classifier chosen in this study seems suitable for prospective prediction. Therefore, we characterized the data in Weka (version 3.8) to make the algorithm fit the data better, the AUC area under the ROC curve of the random forest classifier indicates that the model has a good degree of discrimination, and the real number of filters in the confusion matrix with positive numbers for the training and test sets also indicate that the model has moderate to high reliability for forward-looking predictions. The mangrove secondary metabolite library in the laboratory contained a large number of molecules with different frames, which showed the diversity of candidate compounds. PCA results showed that the mangrove natural product library had a broader space of chemical properties. After the compounds screened by the random forest classifier were introduced into the covalent screening module of Schrödinger Suite 18.4, the control compounds with higher scores were selected for further analysis by comparison with the positive control MRTX849. From the docking results, it seems that the carbon–carbon double bond and the imine group can form a covalent bond with the Csy12 group of KRAS^G12C^ through Michael addition reaction, and these warheads were proved to be feasible in this study. Binding modes and molecular interactions reveal the mode of action of the selected ligands for KRAS^G12C^. The comparison with the positive control showed that the warhead of compound **14** covalently bound to the receptor was similar, which may instruct us to modify it for better effect in the following experiments. Interestingly, the skeleton of compound **44** is similar to that of the positive control MRTX849, which allows compound **44** to have more favorable interactions and better probe itself into the shallow hydrophobic pocket of KRAS^G12C^ to interact with the better receptor combination. Toxicity testing shows that the structure of compound **14** has no components that are toxic to humans, but compound **44** has groups that may be harmful to humans, which is very important for future research. We may be able to optimize the functional groups of the lead compounds to provide them with better performance when targeting KRAS^G12C^. In addition, quantum/molecular mechanics (QM/MM) calculations can be performed on the complexes, and finally, the conformation is selected from the docking simulations [32].

After years of development and calibration, the QM/MM hybrid method has become an indispensable tool for studying the kinetics of various chemical and biochemical processes. QM/MM is mainly used to characterize and study the transition states and activation energies of enzymatic reactions. Conformations computed in this way describe the surrounding environment in more detail. However, the calculations become more complex and not easy to control. We chose the conformation by analyzing the interaction between the molecule and the binding moiety, which focuses not only on the binding mode but also the moiety to be referenced. However, some compounds change mating conformations due to changes in the environment, regardless of environmental influences. Machine learning to resolve inhibitors is an emerging technology that is an important branch of artificial intelligence to extract useful and thematically relevant data when analyzing large samples. In this study, we focused on the classification analysis and principal component analysis of machine learning, allowing the compounds in the data set to pass activity prediction, so that the virtual screening can obtain more drug-like results. At the same time, in the machine learning analysis, if the docking score is more balanced by improving the docking score, it can also accurately filter out potential lead compounds from the virtual screening [33]. Compared with the QSAR analysis under the traditional algorithm [34], the active structure–activity relationship model constructed by machine learning can better fit the data and can have better performance in terms of robustness and prediction accuracy [35]. We, therefore, identified compounds in the mangrove secondary metabolite pool that might target KRAS^G12C^, which exhibited favorable pharmacokinetic properties and docking effects and also received high scores in machine learning models. In the following research, its inhibitory activity can be verified experimentally to better obtain its inhibitory effect in animals and humans.

All in all, in terms of statistical machine learning methods, docking scores, and in silico ADMET studies, the results are satisfactory, indicating that virtual screening strategies combined with machine learning as well as structure-based molecular docking can improve the efficiency and accuracy of screening of target compounds.

## 4. Materials and Methods

### 4.1. Protein Pretreatment

We used the PDB website (https://www.rcsb.org/, accessed on 1 May 2021) to select and download KRAS^G12C^’s structure (PDB id:5F2E) [36], and then imported it into the Schrödinger Suite 18.4 to perform protein processing preparation. Pre-processing took place in the prepwizard module (Schrödinger Inc., New York, NY, USA), flipping pairs of Asn, Gln, and His by 180°. The terminal X angle of the residue was sampled to optimize the hydrogen bond network, and the hydrogen on the hydroxyl and thiols was sampled to optimize the hydrogen bond network. After hydrogen bond optimization, we used impact’s imperf module and OPLS-2005. It also minimizes the structure of the protein; thus allowing the entire structural system to relax. In a protein minimization protocol, including all atoms and pure hydrogen atoms, the conditional criterion for termination was based on the root mean square deviation of the heavy atoms from their initial positions. At the same time, all water molecules were removed under the premise of optimizing hydrogen bonds and retaining the necessary water molecules at the minimum stage. 

### 4.2. Machine Learning 

#### 4.2.1. Data

The dataset constructed to train the machine learning classifier was extracted and queried separately in the ChEMBL database, and the reference protein we chose was ChEMBL2189121. The activity set was defined as compounds with a molecular weight < 1000, and the activity type (Standard type = “IC50”) that detected inhibition had a STANDARD_UNITS value of “NM”. After removal of duplicate structures and no experimentally determined definitive IC50 values, the active set included 98 structures. Standard Relation =“>” for the inactive compound set, which means that the construct did not show any activity at the concentrations used for screening. The inhibitory activity type of the resulting structure was also IC50, and its STANDARD_UNITS was also “NM”. After deduplication, the inactive set contained 72 structures. The electrical properties of the compounds were restored to electrical neutrality for both test and training sets. All machine learning classifiers use L1 regularization to weed out unimportant descriptors. The area under the curve (AUC) of the receiver operator characteristic (ROC) and the number of true positives (TP), true negatives (TN), false positives (FP), and false negatives (FN) were used as metrics for the classification model.

#### 4.2.2. Machine Learning Models

Some of the molecular descriptors in the sample were noisy and irrelevant. We needed to remove them without missing too much information to reduce the likelihood of overfitting. From here, a condition was introduced to remove the unwanted descriptor, which measures the correlation between the descriptor and the sample output by the classifier [37]. For this purpose, we used a feature selection project, which selected the appropriate descriptor in a sample that contains a small amount of information without losing a lot of information. This study used the Rank method in the Select Attributes module in Weka [38] to rank each feature in descending order and then delete the lower-ranked feature. At the same time, the CfsSubsetEval method was used to predict the degree of complexity between each feature and the predicted feature for classification.

#### 4.2.3. QSAR Modeling

The QSAR classification model can reflect the molecular descriptor as a correspondence between the independent and dependent variables, each representing the category of the corresponding sample (KRAS^G12C^ inhibitory activity). Machine learning algorithms can group observations or instances into classes. In structure–activity relationships, it tended to be complex and nonlinear, in which case QSAR modeling had shown excellent performance [39]. Lazypredict Pack (https://github.com/shankarpandala/lazypredict/tree/master, accessed on 12 January 2022) uses a variety of machine learning algorithms to verify which algorithm is better suited for a dataset in Python. The machine learning software Weka (Waikato Knowledge Analysis Environment) [38] version 3.8 was used to perform a random forest algorithm selected by lazy prediction. Weka implemented 10 cross-validations to get the best fit on the training set. 

#### 4.2.4. Principal Component Analysis

Principal component analysis (PCA) was performed on the mangrove secondary metabolite library data set to assess its chemical space. We used the Scikit-Learn 40 (0.22.2) for the PCA algorithm. The pubchem fingerprint reduces the feature dimension to 3. Molecular descriptors and fingerprints were from the Cheminformatics Library Rdkit (1 March 2020).

### 4.3. Covalent Docking

To further screen candidate compounds, the resulting candidate compounds were subjected to covalent docking virtual screening (CovDock-VS) based on the KRAS^G12C^ structure (PDB ID:5F2E). Molecular docking studies were conducted using the Maestro program. The ray structure of the KRAS^G12C^ protein (PDB ID:5F2E) with a resolution of 1.40 Å was selected for covalent docking. The ligands were prepared in Schrödinger’s LigPrep module (Schrödinger Inc., New York, NY, USA). Protonation and ionization states of various stereoisomers, tautomers, and ligands were generated at pH 7.4 using an ionizer. Finally, the energy of the ligand was minimized using the OPLS2005 force field. The LigPrep-generated ligands were docked into the receptor grid in a covalent docking manner and the Michael addition reaction was selected as the reaction equation. The active functional group of the ligand was limited to 5 Å of the active amino acid residue, according to the previously obtained binding sites. A receptor grid with X = 14.9, Y = 10.5, and Z = 14.3 was prepared. The energy was minimized after docking, and each ligand outputs up to three optimal poses. Unless otherwise noted, all docking results were visually screened and conformations with the best docking scores were retained. CovDock used Cys12 as a covalently mated nucleophilic residue that conjugates to CovDock’s preset alkyne hydrocarbons (carbonyl activation). Ligands with reactive functional groups in the range of 5 Å form covalent bonds specified by the reaction. Ligands were selected and ranked based on the Glide score of the reaction complex binding pattern [40]. To ensure the accuracy of the docking protocol, we chose the superposition module in maestro to calculate the docking RMSD between the ligand’s pose and crystal coordinates, while using MRTX849 as a control.

### 4.4. ADME

ADME’s analysis of the pharmacodynamics of this pharmaceutically acceptable small molecule was of great significance. The Swiss ADME Server (http://www.swissadme.ch/, accessed on 17 December 2021) evaluates lead compounds retained after machine learning and covalent docking screening. This was described based on the specification SMILES [41]. The ADME properties of the selected compound were calculated by the website. The main relevant parameters such as pharmacokinetic properties and drug solubility were taken into account. The observed attribute values are shown in Table 3. 

### 4.5. Pharmacophore Modeling and Matching Validation 

By using the Discovery Studio platform (Discovery Studio 4.5, Accelrys, Co., Ltd., 175 Wyman Street, 02451 WALTHAM, MA, USA), we spatially aligned three known positive compounds and generated 10 hypothetical pharmacophore models based on molecular common characteristics. We selected hydrogen bond receptors, hydrogen bond donors, and hydrophobicity features as model pharmacodynamic features. The minimum distance was set between pharmacodynamic features within the model to 2.97 Å and the best conformation method was applied to generate the potential conformation of the positive compound. According to the pharmacophore ranking score given by the platform, the optimal pharmacophore was selected to match the two candidate molecules to assess whether the candidate molecules were consistent with the common pharmacodynamic characteristics of known inhibitor molecules.

### 4.6. Molecular Dynamics (MD) Simulation

After docking, an MD simulation of the compounds **14** and **44** with KRAS^G12C^ was used to check the stability of the compound in the binding bag. Then, the GROMACS 2019.1 package [42], amber 99sb-ildn force field (https://www.gromcs.org/About_Gromacs, accessed on 26 December 2021), and single point charge (SPC216) model were used for molecular dynamics simulations of 100 ns. To guarantee the total charge neutrality of the simulated system, a corresponding number of sodium ions are added to replace the water molecules in the system to produce a solvent cartridge of appropriate size. Then, the periodic boundary condition (PBC) was applied in the three directions of the system [43]. Using the amber99sb-ildn force field, the force field parameters obtained for the entire atom can be found on the Acpype website [44] (https://www.bio2byte.be/acpype/, accessed on 29 December 2021). The first pass (EM) minimizes the energy of the entire system at 50,000 steps below 300 K. Then, through MD simulations with position constraints, collected by NVT (constant particle count, volume, and temperature), and finally by NPT (constant particle number, pressure, and temperature) [45]. In addition, we balanced enzymes, ligand molecules, and solvents. Among them, we carried out non-standardized residual treatment of covalent bonds in the system. 

### 4.7. MM-PBSA

Poisson Boltzmann Surface Area is open-source software, and g_mmpbsa was primarily used to calculate the free energy of binding between the receptor and the inhibitor after MD [46]. As a scoring function, MM-PBSA has been used in computational methods for drug design. In this study, MM-PBSA was used to determine the binding free energy of KRAS^G12C^ with molecules 44 and 14, respectively. 

The following Equation (2) describes the binding free energy:

(2) $$G_{binding}=G_{complex}-\left(G_{protein}+G_{ligand}\right)$$

The free energy of the protein–inhibitor complex was represented by the *G__complex_*_,_ the free energy of the protein in the solvent is represented by the *G__protein_*_,_ and the free energy of the inhibitor in the solvent is represented by the *G__ligand_*_._

## 5. Conclusions

In summary, marine natural products are an important source of lead compounds, especially mangroves and their secondary metabolites have many potential antitumor lead compounds. In the present study, we constructed a random forest classifier with excellent discriminatory power and sensitivity and used it to predict mangrove-derived compounds with potential KRAS^G12C^ inhibitory activity. Subsequently, further covalent docking and MM-GBSA analysis results confirmed the stable binding ability of two mangrove-derived compounds **14** and **44**. To investigate the commonalities in the potency of our two selected mangrove compounds and previously reported KRAS^G12C^ inhibitors, a pharmacophore model based on molecular common features was also used to further extend our study and corroborate the potential of both compounds to inhibit KRAS^G12C^. Next, our work is focused on improving the biochemical and pharmacological profiles of mangrove secondary metabolite compounds **14** and **44** through further medicinal chemistry work and structural studies. Although the development of KRAS^G12C^ inhibitors is still considered a challenging task in the field of drug discovery and development, our work expands new horizons for this field.

## Figures and Tables

**Table 1:** Prediction values for the training set for the random forest classifier.

Class	Precision	Recall	F-Measure	MCC
Active	0.946	0.993	0.969	0.825
Inactive	0.962	0.758	0.847	0.825
Weighted Avg	0.949	0.949	0.946	0.825

**Table 2:** Predicted values for the test set of a random forest classifier.

Class	Precision	Recall	F-Measure	MCC
Active	0.795	0.939	0.861	0.289
Inactive	0.600	0.273	0.375	0.289
Weighted Avg	0.746	0.773	0.740	0.289

**Table 3:** Docking RMSD between ligand pose and crystal coordinates for compounds with better docking results than positive controls.

Name	2D Structure	RMSD	Docking Score
**8**	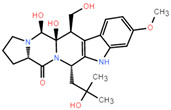	8.7699	−6.518
**14**	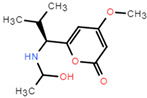	9.1083	−6.707
**15**	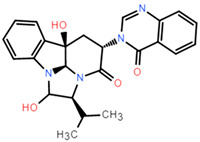	8.3822	−6.432
**31**	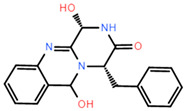	10.9337	−6.52
**44**	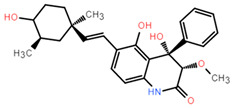	7.9196	−6.916
**75**	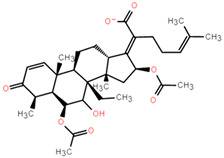	8.4722	−6.65
**102**	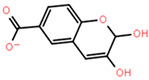	12.8430	−7.618
**127**	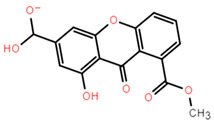	12.7782	−7.701

**Table 4:** ADME properties of ligands selected from the Marine Natural Products Library.

Molecule	MW	Rotatable Bonds	H-Bond Acceptors	H-Bond Donors	ESOL Log S	TPSA	WLOGP	GI Absorption	log Kp (cm/s)
14	241.28	5	5	2	−1.93	71.7	0.95	High	−7.02
31	323.35	2	4	3	−2.69	85.16	−0.38	High	−7.58
44	437.53	4	5	4	−4.66	99.02	3.01	High	−6.6
127	315.25	3	7	2	−3.04	120.03	1.54	High	−7.11

**Table 5:** Feature composition and ranking score of 10 pharmacophore hypothesis models generated based on common features of positive compounds.

ID	Features	Rank	Direct Hit	Partial Hit	Max Fit
1	HHHHAAA	52.937	111	000	7
2	HHHHAAA	52.338	111	000	7
3	HHHHAAA	51.858	111	000	7
4	HHHHHAA	51.669	111	000	7
5	HHHHHAA	51.530	111	000	7
6	HHHHAAA	51.409	111	000	7
7	HHHHHAA	51.359	111	000	7
8	HHHHHAA	51.352	111	000	7
9	HHHHAAA	51.242	111	000	7
10	HHHHAAA	51.236	111	000	7

## Data Availability

The data used to support the findings of this study are included within the article.
